# Supplementary material for: Four weeks versus six weeks of ampicillin plus ceftriaxone in Enterococcus faecalis native valve endocarditis: A prospective cohort study
Source: PLoS One. 2020 Aug 3;15(8):e0237011. doi: 10.1371/journal.pone.0237011 (PMC7398509; doi:10.1371/journal.pone.0237011)
Supplement: Attachment — Submitted filename: Response to reviewers - PONE-D-20-05909R1.docx [file pone.0237011.s001.docx]

**Journal Requirements**

We have tried to all meet PLOS ONE's requirements. The online system on the manuscript submission has not allowed us to enter the funding information correctly. Please update our funding statement to include: 'This work was supported in part by the “Fondo de Investigaciones Sanitarias” (FIS) grant 17/01251 from the “Instituto de Salud Carlos III”, Madrid, Spain awarded to JMM. JMM received a personal 80:20 research grant from the Institut d’Investigacions Biomèdiques August Pi i Sunyer (IDIBAPS), Barcelona, Spain, during 2017–19. JMP was member of the Endocarditis Team of the Hospital Clinic of Barcelona, Spain when this project was approved by the GAMES Steering Committee.'

All authors declare no potentially competing interests that occurred within 5 years of conducting the research under consideration nor preparing the article for publication.

**Reviewer**

1. Statistical issue: p8, 202-204. It is stated that continuous variables will be reported as a median with IQR and that a student's t-test will be used to compare these values. If you report values as a median with IQR, it means that your data was not normally distributed. Did you check if the continuous variables had a normal distribution? In that case, it is not correct to use a student's t-test and a non-parametric test should be used.

We appreciate the reviewer's comment. The quantitative variables studied did not present a normal distribution, so it was necessary to use the Mann-Whitney test. Accordingly, the paragraph referring to statistical analysis has been modified.

2. Statistical issue: p8, 204. The aim of this study is to test whether the clinical outcome of patients that received AC for 4 weeks is similar to those that received AC for 6 weeks. Student t-tests are made to test if there is a significant difference, not to test equivalence. Did you perform an equivalence test or can this be conducted?

In fact, we did not use the Student t-tests to ascertain whether there was a difference between the two groups. What we intended to do was to check whether the prognosis of the patients (assessed by the rate of disease recurrence or mortality) was similar with both therapeutic regimes. We considered that the most appropriate way to check that the two regimes were not different in our patients was to use a qualitative test such as Fisher's.

3. p3, 100-101. In the abstract you mention that patients that received AC for 4 weeks had a longer duration of symptoms (21 days) than patients that received AC for 6 weeks (7 days). In table 1, the values are the opposite. Is this a mistake? Should be corrected, because it is concluded that treatment with AC for 4 weeks may be considered in patients with a shorter duration of symptoms.

In effect, it is a mistake. Patients who were treated for 4 weeks had a shorter duration of symptoms before admission. As noted in previous research, it has traditionally been considered that treatment of this infection should be at least 6 weeks in patients with longer duration of symptoms or with perivalvular abscess

4. Does severity of symptoms correlate with a worse outcome? For example, perivalvular abscesses, vegetation size, valve perforation, ...

A sentence has been added about the absence of influence on the prognosis (infection recurrence and mortality) of echocardiography findings as the presence of perivalvular abscess, valve perforation or a vegetation size larger than 10 mm

5. Data represented in table 1 will be more clear if this table is divided in to two tables: one with the baseline characteristics and another one with the data on outcome.

Table 1 has been broken down into 2 tables on the advice of the reviewer
